# 

**Published:** 1898-12

**Authors:** 


### CONTENTS:

### SELECTIONS.

Article I.—On Structural Changes in Human Enamel—With Special Reference to Clinical Observations on Hard and Soft Enamel. J. Leon Williams, D. D. S., L. D. S., F. R. M. S .......... 337
" II.—The Dentist. Dr. B. F. Shepherd .......... 349
" III.—Secrets and Patents Versus Professional Progress. J. Morgan Howe, M. D. S., M. D .......... 354
" IV.—The Prolongation of Nitrous Oxid Anesthesia. Herbert J. Paterson, M. A., M F. Cant., F. R C. S.... 361
" V.—Frequency of Dental Caries in Children .......... 364
" VI.—The Physical Properties of Dental Amalgams. The late Prof. T. B. Hitchcock, M. D., D. M. D .......... 366
" VII.—Reciprocity .......... 368
" VIII.—Display of Gold in Teeth .......... 371
" IX.—An Important Dento-Legal Function. A. I. Benedict, A. M., M. D .......... 372

### BIBLIOGRAPHICAL.

A Dictionary of Dental Science .......... 375

### MONTHLY SUMMARY.

Committees of the National Dental Association .......... 376

Hemorrhage After Extraction .......... 376

Natural Law and its Penalties .......... 377

Filling with Gold over Cement .......... 378

Pain and the Surgeon .......... 380

National School of Dental Technics .......... 381

A Good Cement .......... 381

Preservation of Ether .......... 382

Plaster Impressions and Models .......... 382

A Cheap Method of Crowning .......... 382

Mercury to Purify .......... 383

Treating Foul Pulp-Canals .......... 383

Reflected Light for the Operating Chair .......... 383

To Solder Cap on Gold Crown .......... 383

Fitting Porcelain Inlays .......... 384

Sandpaper your Joints .......... 384

Carious Dentin .......... 384

